# Transport of Methylmercury and Inorganic Mercury to the Fetus and Breast-Fed Infant

**DOI:** 10.1289/ehp.7856

**Published:** 2005-06-15

**Authors:** Karolin Ask Björnberg, Marie Vahter, Birgitta Berglund, Boel Niklasson, Mats Blennow, Gunilla Sandborgh-Englund

**Affiliations:** 1 Division of Metals and Health, Institute of Environmental Medicine, Karolinska Institutet, Stockholm, Sweden; 2 Division of Pediatrics and; 3 Department of Obstetrics and Gynecology, Karolinska University Hospital, Huddinge, Sweden; 4 Division of Dental Biomaterials, Institute of Odontology, Karolinska Institutet, Huddinge, Sweden

**Keywords:** breast-feeding, breast milk, human, infant exposure, inorganic mercury, methylmercury, pregnancy

## Abstract

It is well established that methylmercury (MeHg) and mercury vapor pass the placenta, but little is known about infant exposure via breast milk. We measured MeHg and inorganic mercury (I-Hg) in blood of Swedish mothers (*n* = 20) and their infants, as well as total mercury (T-Hg) in breast milk up to 13 weeks postpartum. Infant blood MeHg was highly associated with maternal blood MeHg at delivery, although more than twice as high. Infant MeHg decreased markedly until 13 weeks of age. Infant blood I-Hg was associated with, and about as high as, maternal blood I-Hg at delivery. Infant I-Hg decreased until 13 weeks. In breast milk, T-Hg decreased significantly from day 4 to 6 weeks after delivery but remained unchanged thereafter. At 13 weeks, T-Hg in breast milk was associated with infant MeHg but not with maternal MeHg. Conversely, T-Hg in breast milk was associated with maternal I-Hg but not with infant I-Hg. From the findings of the present study in which the exposure to both MeHg and I-Hg was low, we conclude that the exposure to both forms of mercury is higher before birth than during the breast-feeding period, and that MeHg seems to contribute more than I-Hg to infant exposure postnatally via breast milk.

People are exposed to methylmercury (MeHg) mainly through consumption of predatory fish species, and to inorganic mercury (I-Hg) mainly through release of mercury vapor (Hg^0^) from amalgam fillings. Both MeHg and I-Hg are neurotoxic, especially for the developing brain [National Research Council (NRC) 2000], and it is well documented that both MeHg and Hg^0^ readily pass the placenta [Ask et al. 2002; NRC 2000; Vahter et al. 2000; World Health Organization (WHO) 1991]. In general, MeHg concentrations in cord blood are almost twice those in maternal blood (Sakamoto et al. 2004; Stern and Smith 2003; Vahter et al. 2000), probably due to transport via the neutral amino acid carrier (Kajiwara et al. 1996). Blood I-Hg concentrations are about the same in mother and newborn (Vahter et al. 2000).

The brain is sensitive to chemical assaults also postnatally (Rice and Barone 2000) but little is known about infant mercury exposure via breast milk. Breast-feeding has many benefits, and breast milk is the best source of nutrition for infants (Gartner et al. 1997; Oddy 2002; Pronczuk et al. 2004). However, it may also be a source of environmental contaminants (Anderson and Wolff 2000; Dorea 2004). Findings of decreasing concentrations of I-Hg in maternal blood during breast-feeding indicate that I-Hg is excreted in breast milk (Vahter et al. 2000). This is supported by reported correlations between total mercury (T-Hg) in milk and plasma, believed to contain mainly I-Hg (Skerfving 1988), and between I-Hg in milk and I-Hg in whole blood (Oskarsson et al. 1996). Also, T-Hg in milk correlated with number of amalgam fillings, but not with fish consumption (Oskarsson et al. 1996). However, another study found correlations to both (Drexler and Schaller 1998). Both human and animal studies indicate that the infant may be exposed to MeHg via breast milk (Grandjean et al. 1994; Nordenhall et al. 1998; Sundberg et al. 1991). However, to what extent I-Hg and MeHg in breast milk are taken up by the child is not known. By speciating the blood concentrations of MeHg and I-Hg at birth and during breast-feeding in relation to the concentrations in maternal blood and breast milk, we aimed in the present study to clarify the transport of MeHg and I-Hg during breast-feeding and infant exposure through breast milk.

## Materials and Methods

In 2001 we recruited 20 women at delivery at the Huddinge University Hospital, using a convenience sample. The midwives involved in the project informed women registering at the delivery clinic about the study. No particular exclusions were made, but emergency cases were not included. The participation was voluntary, and only a few of the approached women chose not to participate. There were no incentives. Reason for nonparticipation was the inconvenience of sampling.

We obtained information about smoking and alcohol consumption from the antenatal care centers. Four women reported that they did consume alcohol, although seldom, and two women reported smoking, one of them quitting during pregnancy. The women had normal and healthy pregnancies. However, one woman developed late preeclampsia (week 39), and one had premature delivery (week 35). Two women delivered by cesarean section. One infant was born with cleft palate, and one with functional ileal obstruction. The general health and growth of all infants were good at the last sampling occasion.

We collected blood samples from the mothers (antecubital vein) and infants at the time of delivery and at approximately 4 days (in connection with the phenylketonuria test; range, 3–6 days) and at 13 weeks (on average, 94 days; range, 86–115 days) after delivery. Infant blood was collected from the umbilical artery at delivery, the femoral vein at 4 days, and the hand vein at 13 weeks. The mothers collected breast milk samples at approximately 4 days (colostrum; range, 2–5 days), 6 weeks (46 days; range, 34–59 days), and 13 weeks (88 days; range, 77–101 days) after delivery. Because of the known variability in breast milk composition, the women were asked to collect three different samples (> 5 mL) at 6 and 13 weeks, respectively; to register the time when the sample was taken; and to specify whether the sample was collected at the beginning, middle, or end of the feeding. A total of 15 women collected samples on all three occasions.

At delivery, the women filled out a questionnaire concerning fish consumption, vaccinations, and dental care during the preceding 6 months. At the 13-week appointment, the women completed a similar questionnaire that also included information about breast-feeding and the use of infant formula. Six women reported use of infant formula in combination with breast-feeding at 13 weeks. In addition, a dentist recorded the number of amalgam-filled surfaces. We obtained informed consent from the women, and the Ethics Committee at Karolinska Institutet approved the study.

We analyzed T-Hg and I-Hg in blood by alkaline solubilization/reduction and cold-vapor atomic fluorescence spectrophotometry (CVAFS; Merlin, PSA 10.023; P.S. Analytical Ltd., Orpington, Kent, UK) as previously described (Vahter et al. 2000). The concentration of organic mercury was calculated as the difference between T-Hg and I-Hg. We assume that essentially all of the organic mercury in blood was in the form of MeHg, because the only other known exposure sources of organic mercury compounds in Sweden are a few vaccines containing thimerosal (a preservative containing ethylmercury). None of the women had received any vaccines during the pertinent period, and none of the infants had received vaccines containing thimerosal. The use of thimerosal in vaccines for infants and children in Sweden stopped in the early 1990s. In breast milk, we analyzed T-Hg by CVAFS (Merlin, PSA 10.003; P.S. Analytical Ltd.) (Sandborgh-Englund et al. 1998) after acid microwave digestion. Breast milk samples of 1.0 mL were mixed with 1.5 mL concentrated HNO_3_ (suprapure) and digested in a microwave oven (model MDS-2000; CEM-Innovators Microwave Technology, Matthews, NC, USA). We were not able to speciate mercury in breast milk, although we tried different methods. The main problem was the solubilization step. Digestion of breast milk samples in a microwave oven enabled us to determine T-Hg. Because the concentrations were very low, we made no further attempts to speciate mercury in milk. All utensils used were checked to be free from mercury contamination.

The limit of detection (LOD; 3 × SD of the reagent blanks) in blood varied between 0.04 and 0.09 μg/L for T-Hg and between 0.03 and 0.08 μg/L for I-Hg. The LOD for T-Hg in breast milk varied between 0.03 and 0.07 μg/L. One-third (*n* = 40) of the blood samples (from 10 of the women and 15 of the infants) had I-Hg concentrations below the LOD, and two breast milk samples (from two different women) had T-Hg concentrations below the LOD.

Results of the analytical quality control are presented in Table 1. There are no recommended values for I-Hg in the Seronorm reference blood samples (Nycomed Co., Oslo, Norway), but the values obtained for I-Hg were in good agreement with our previous analytical runs of the same Seronorm sample (Seronorm 404107: 0.53 ± 0.06 μg I-Hg/L, *n* = 21; Seronorm 404108: 6.2 ± 0.59 μg I-Hg/L, *n* = 27). Repeated analysis of a MeHg standard containing 0.4 μg Hg/L during blood analysis gave a recovery of 99 ± 5%. Repeated analysis of standard solutions containing 1.25 and 2.5 ng T-Hg during breast milk analysis gave a recovery of 100 ± 4%. Seven breast milk samples from one woman, sampled at different occasions, were replicated in three separate analyses. The coefficient of variation varied between 2 and 16%.

### Statistics.

The mercury concentrations were not normally distributed (skewness and Shapiro-Wilk tests of normality). To test for correlations between variables, we used Pearson correlation (*r*) whenever the requirements for normally distributed residuals were met; otherwise, we used Spearman correlation (*r*_S_). We used Wilcoxon and Kruskal-Wallis nonparametric tests to test for differences between variables and groups.

For evaluation of variation in mercury concentrations over time, we log-transformed the data to be able to use analysis of variance (ANOVA) for repeated measures with one within-subject factor (time) with three levels. To test for differences in mercury concentrations between specific time points, we used contrast analysis.

Statistical analyses were conducted mainly with SPSS (version 12.0.1 for Windows; SPSS Inc., Chicago, IL, USA). The influence of sampling in relation to feeding (beginning, middle, or end) was evaluated using the Mixed procedure in SAS System 8.2 (SAS Institute Inc., Cary, NC, USA). We computed two-way repeated-measures ANOVA with feeding (beginning, middle, and end) and week (6 and 13) as the within-subjects variables. Statistical significance level was set to *p* < 0.05.

Although values below LOD have larger uncertainty than do those above, analytical results below LOD were not rejected or changed so that distributions would not be distorted. In the evaluation of correlations between T-Hg in breast milk and the different forms of mercury in blood, we used mean values of all breast milk samples from each woman taken at 6 weeks and 13 weeks, respectively.

## Results

A summary of study characteristics is presented in Table 2. Minor dental treatment was reported by seven women during pregnancy and two women during breast-feeding. They did not have higher blood mercury concentrations compared with those who did not have dental treatment. The blood mercury concentration of children given infant formula in combination with breast-feeding (*n* = 6) did not differ from those exclusively breast-fed. The women’s total fish intake was similar during pregnancy as during breast-feeding (*p* = 0.25). No woman reported intake of freshwater fish during pregnancy or breast-feeding. Information on consumption of predatory marine fish was not explicitly asked for.

**Figure 1:**
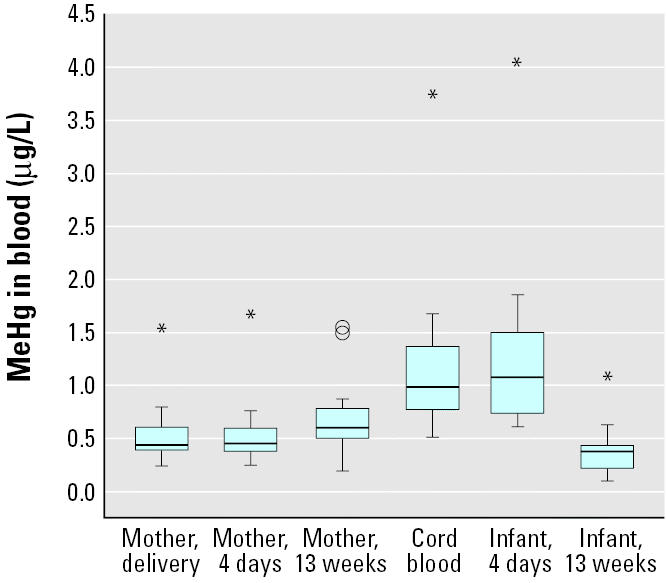
The concentrations of MeHg in maternal and infant blood at delivery and 4 days and 13 weeks postpartum (*n* = 20). Box length illustrates the interquartile range; line is the median. Circles are cases with values 1.5–3 box lengths from the upper or lower edge of the box, and asterisks are cases with values > 3 box lengths from the upper or lower edge of the box.

**Figure 2:**
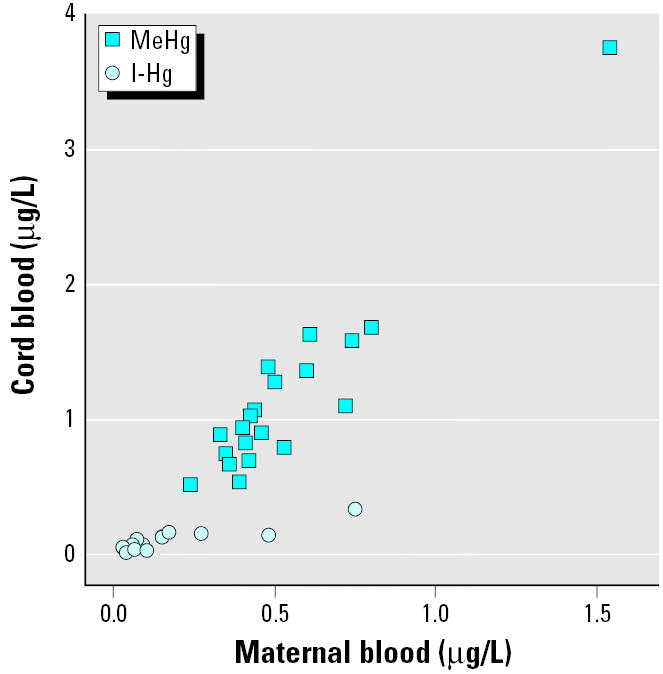
The associations between concentrations in cord blood and maternal blood for MeHg (*r* = 0.95; *p* < 0.001) and I-Hg (*r*_S_ = 0.77; *p* < 0.001).

As shown in Figure 1, maternal blood MeHg increased from delivery (median, 0.45 μg/L; range, 0.24–1.5 μg/L) to 13 weeks postpartum (median, 0.60 μg/L; range, 0.20–1.6 μg/L; *p* = 0.01). MeHg in maternal blood was associated with MeHg in cord blood (median, 0.99 μg/L; range, 0.52–3.8 μg/L; Figure 2) and in infant blood at 4 days (median, 1.1 μg/L; range, 0.62–4.4 μg/L; *r* = 0.95; *p* < 0.001), although the cord and infant blood concentrations were more than twice as high as those in maternal blood. Infant blood MeHg decreased (*p* < 0.001) from 4 days to 13 weeks after birth (median, 0.38 μg/L; range, 0.10–1.1 μg/L; Figure 1). We did not find any significant associations between reported fish consumption and maternal or infant blood MeHg.

**Figure 3:**
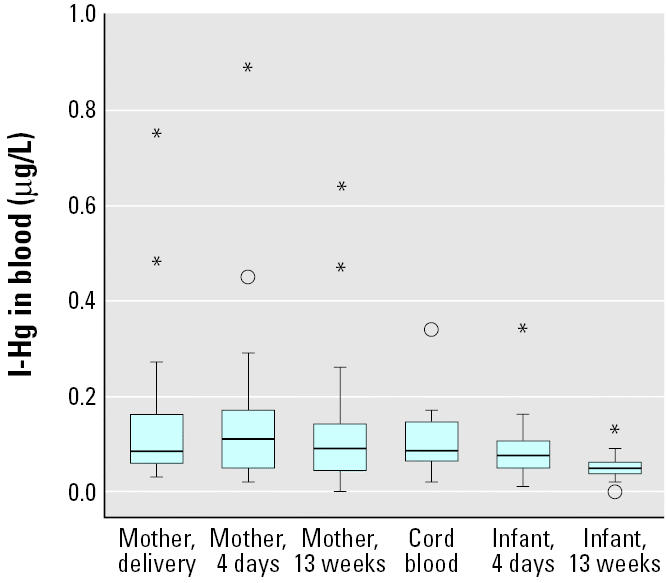
The concentrations of I-Hg in maternal and infant blood at delivery and 4 days and 13 weeks postpartum (*n* = 20). Box length illustrates the interquartile range; line is the median. Circles are cases with values 1.5–3 box lengths from the upper or lower edge of the box, and asterisks are cases with values > 3 box lengths from the upper or lower edge of the box.

As shown in Figure 3, maternal blood I-Hg was about the same at delivery (median, 0.09 μg/L; range, 0.03–0.75 μg/L) as at 13 weeks postpartum (*p* = 0.78). Infant blood I-Hg (median, 0.09 μg/L; range, 0.02–0.34 μg/L) was similar to maternal concentrations at birth and was significantly associated with maternal blood I-Hg both at birth (Figure 2) and at 4 days (*r*_S_ = 0.51; *p* = 0.02). Infant blood I-Hg decreased (*p* = 0.001) until 13 weeks of age (median, 0.05 μg/L; range, 0–0.13 μg/L). Maternal blood I-Hg correlated significantly with the number of amalgam-filled surfaces at delivery (*r*_S_ = 0.55; *p* = 0.01).

**Figure 4:**
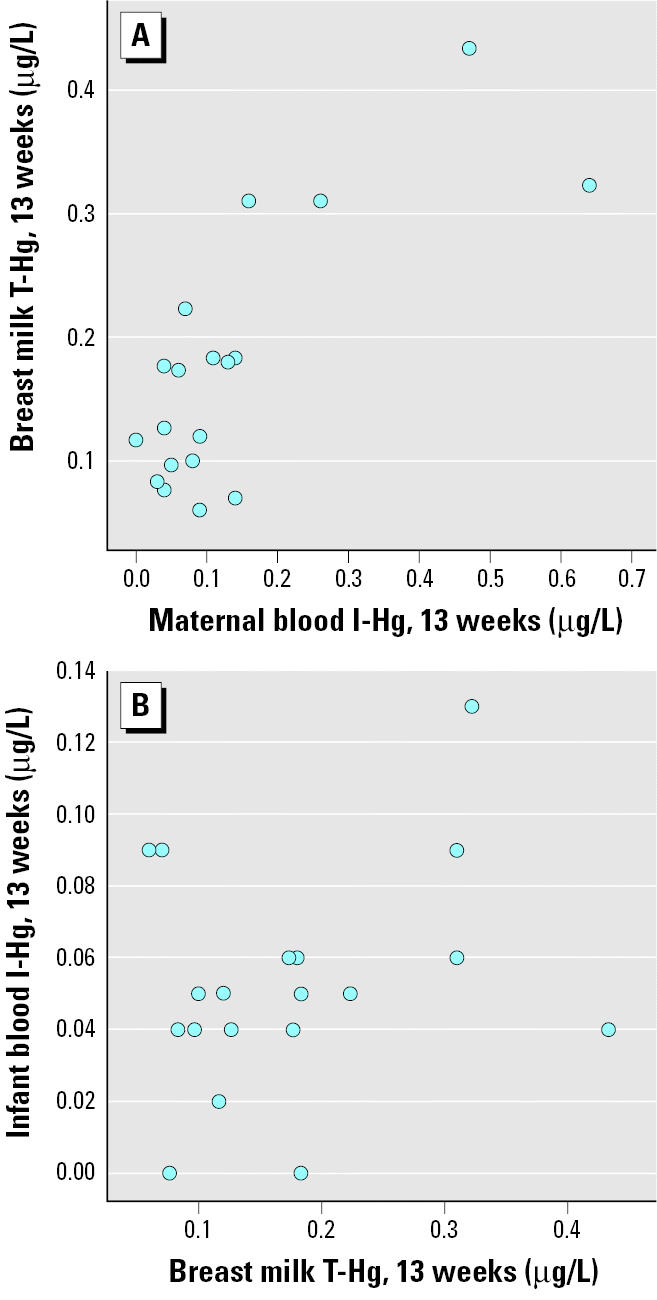
The associations between T-Hg in breast milk and I-Hg in maternal blood (*A*; *r*_S_ = 0.61; *p* = 0.006) and infant blood (*B*; *r*_S_ = 0.17; *p* = 0.50) at 13 weeks after birth.

**Figure 5:**
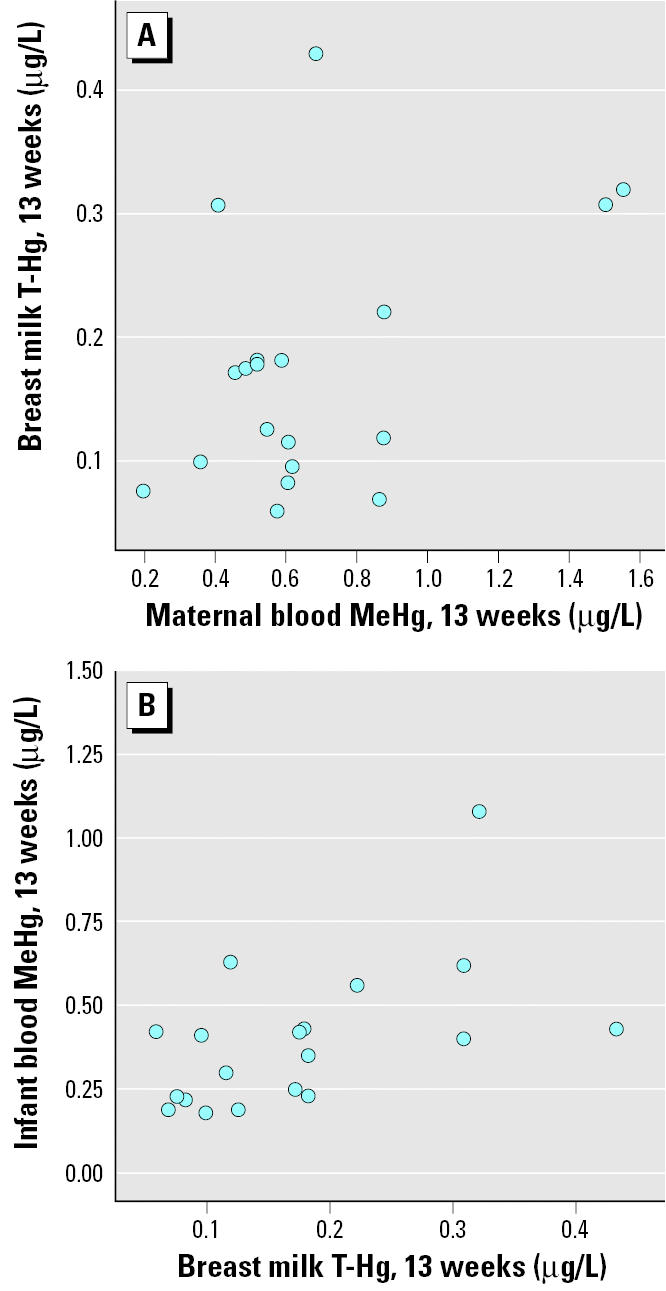
The associations between T-Hg in breast milk and MeHg in maternal blood (*A*; *r*_S_ = 0.26; *p* = 0.28) and infant blood (*B*; *r*_S_ = 0.55; *p* = 0.01) at 13 weeks after birth.

**Figure 6:**
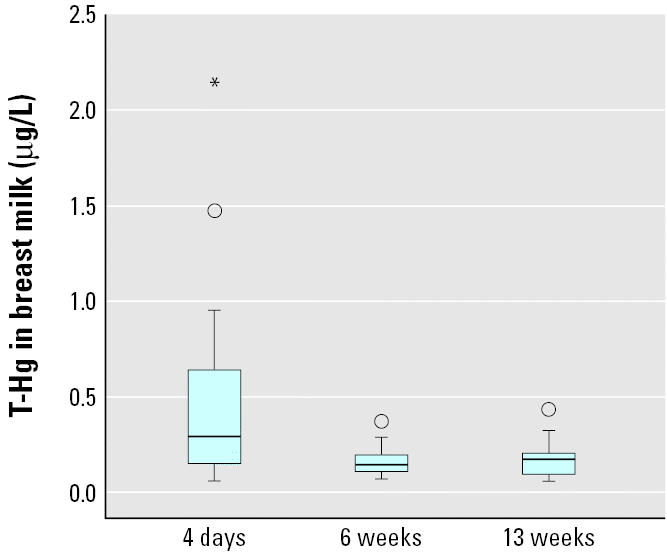
The concentrations of T-Hg in breast milk at 4 days (colostrum; *n* = 19), 6 weeks (*n* = 20), and 13 weeks postpartum (*n* = 19). Box length illustrates the interquartile range; line is the median. Circles are cases with values 1.5–3 box lengths from the upper or lower edge of the box, and asterisks are cases with values > 3 box lengths from the upper or lower edge of the box.

As shown in Figure 4, T-Hg in breast milk at 13 weeks correlated significantly to maternal blood I-Hg but not to infant blood I-Hg. Conversely, T-Hg in breast milk at 13 weeks correlated significantly to infant blood MeHg (Figure 5) but not to maternal blood MeHg. T-Hg in breast milk (Figure 6) decreased significantly (*p* < 0.001) from day 4 (colostrum; median, 0.29 μg/L; range, 0.06–2.1 μg/L) to 6 weeks postpartum (mature milk; median, 0.14 μg/L; range, 0.07–0.37 μg/L) but remained unchanged thereafter. T-Hg in milk increased significantly with time during each feeding session at 6 weeks (*n* = 15; *p* < 0.001). The median concentrations were 0.12 μg/L (range, 0.04–0.31 μg/L) in the first milk that was pumped out, 0.15 μg/L (range, 0.07–0.32 μg/L) about halfway through the feeding session, and 0.18 μg/L (range, 0.07–0.49 μg/L) at the end. This change was less pronounced at 13 weeks.

T-Hg in breast milk correlated with the number of amalgam-filled surfaces at 4 days (*r*_S_ = 0.49; *p* = 0.04) and 6 weeks (*r*_S_ = 0.44; *p* = 0.05). We did not find any significant associations between T-Hg in breast milk and reported fish consumption. Parity did not affect the concentrations of T-Hg in breast milk.

## Discussion

The present study clearly demonstrates that infant exposure to MeHg and I-Hg via breast-feeding is low compared with late fetal exposure. For both MeHg and I-Hg, the concentrations in maternal blood and cord blood at delivery were highly associated, which is in accordance with previous studies (Stern and Smith 2003; Vahter et al. 2000). Because the women of the present study had few amalgam fillings and a limited consumption of fish, especially fish species potentially high in MeHg, they also had low mercury exposures. Apparently, their compliance with the recommendation that pregnant and breast-feeding women avoid consumption of certain fish species (Livsmedelsverket 2004) was good.

Exposure to MeHg and I-Hg in breast-fed infants is largely unknown. Animal studies suggest that both MeHg and I-Hg are transported from plasma to breast milk bound to serum albumin, with I-Hg also bound to casein (Sundberg et al. 1999). Besides the binding to albumin and casein in breast milk, the transport is also affected by the distribution in maternal blood. About 65% of I-Hg but only about 10% of MeHg in whole blood is present in plasma (Kershaw et al. 1980) and thus available for transport to breast milk.

We found no significant association between MeHg in maternal blood and mercury in breast milk, which is in contrast to a recent Japanese study (Sakamoto et al. 2002). Probably this was due to the lower MeHg exposure and higher I-Hg exposure (as Hg^0^ via amalgam fillings) in the present study. Unfortunately, we were not able to speciate mercury in breast milk. Based on the reported milk-to-plasma ratios of about 0.2 for MeHg (Sakamoto et al. 2002; Sundberg et al. 1998) and 0.6–1.0 for I-Hg (Oskarsson et al. 1996; Sundberg et al. 1998), it seems that I-Hg is more easily transported to breast milk than is MeHg. Also, the association between I-Hg in maternal blood and mercury in breast milk indicated transport of I-Hg into milk. The absorption of Hg^2+^ in the gastrointestinal tract is known to be low (Sandborgh-Englund et al. in press; WHO 1991), but it has been suggested to be higher in infants than in adults (Clarkson 1992). However, we found that infant blood I-Hg concentrations declined from birth to 13 weeks of age (Figure 3) and there was no association between mercury in breast milk and I-Hg in infant blood. Thus, our result raises the question as to what extent I-Hg in milk is absorbed from the gastrointestinal tract in the infant.

On the other hand, we found that mercury in breast milk correlated with MeHg in infant blood at 13 weeks. This might be explained by the fact that the small amount of MeHg that passes from maternal plasma to breast milk is almost completely taken up by the infant [about 95% is absorbed in the gastrointestinal tract (WHO 1990)]. In this way, there is a small exposure of the breast-fed infant to MeHg via breast milk. Still, infant blood MeHg decreased markedly until 13 weeks of age (Figure 1). The decline, which is in line with previous findings (Sakamoto et al. 2002; Sandborgh-Englund et al. 2001), can be explained partly by the rapid increase in body weight, and partly by a decrease in hematocrit (Ciba-Geigy 1984) due to the exchange from fetal to adult hemoglobin. It is generally believed, based on results from animal studies, that excretion of MeHg (which takes place mainly via feces) by the breast-fed newborn is limited, because demethylating bacteria in the gastrointestinal tract become first established after weaning (Rowland et al. 1983). Unless it is demethylated, MeHg is reabsorbed via the enterohepatic circulation. With limited excretion of MeHg in the infant, one would not expect a marked decline in blood MeHg concentrations during the first 3 months of life as found in the present study. Studies of the total postnatal excretion of MeHg are needed to confirm that the prenatal accumulated MeHg actually may be excreted during the first postnatal weeks.

Both volume and composition of breast milk change over time, during a feeding session, during the course of the day, and during the breast-feeding period (Mitoulas et al. 2002). In the present study we found T-Hg levels approximately twice as high in colostrum as in mature breast milk. Probably the higher concentration of albumin in colostrum compared with that in mature milk (Neville et al. 1983) enables transport of both forms of mercury. However, the increasing volume of breast milk during the breast-feeding period (Kunz et al. 1999) contributes to a dilution of the concentrations of mercury in mature milk. Interestingly, we also found an increase in mercury concentrations in breast milk during the same feeding, which could be explained by an increase in casein concentrations during the feeding (Hytten 1954).

Given the findings of the present study, we conclude that the child’s exposure to MeHg and I-Hg is much higher before birth than during breast-feeding. Exposure of infants to MeHg may also occur postnatally through breast milk, depending on maternal plasma MeHg concentrations. This underlines the importance of dietary recommendations to pregnant and breast-feeding women not to eat MeHg-contaminated fish. However, because the present study was carried out in a small group with low exposure to both MeHg and I-Hg, further studies of the different forms of mercury in breast milk and infant uptake are warranted in populations with higher exposure (Barbosa and Dórea 1998).

## Figures and Tables

**Table 1:** Results from analytical quality control of T-Hg and I-Hg in blood and T-Hg in breast milk (μg/L).

Quality control	Species	Recommended value	Obtained value (*n* = 6)	Coefficient of variation (%)
Breast milk				
SRM-1549	T-Hg	0.3 ± 0.2	0.3 ± 0.1	25
Blood				
Seronorm	T-Hg	2.2–3.3	2.4 ± 0.2	8.6
404107	I-Hg	—	0.55 ± 0.03	5.0
Seronorm	T-Hg	6.7–8.4	8.0 ± 0.3	3.1
404108	I-Hg	—	6.5 ± 0.2	2.6

Abbreviations: —, no data; SRM, standard reference material (National Institute of Standards and Technology, Gaithersburg, MD, USA).

**Table 2:** Subject characteristics (*n* = 20).

Characteristic	Mean	Minimum	Maximum
Age (years)	31	24	37
Body mass index	26	21	36
Weight gain during pregnancy (kg)	15	–1	24
Parity (*n*)	1	0	3
Gestational length (weeks)	39	35	43
Fish intake, pregnancy (times/month)	2.0	0	8
Fish intake, breast-feeding (times/month)	2.4	0	8
Amalgam-filled surfaces (*n*)	5	0	24
Infant birth weight (g)	3,666	2,720	4,505
